# Anatomical, functional, and patient-reported outcomes following anterior urethroplasty. Can we predict when and why are patients with anatomical recurrences requiring reinterventions?

**DOI:** 10.1007/s00345-026-06335-y

**Published:** 2026-03-11

**Authors:** Maite Miqueleiz Legaz, Felix Campos-Juanatey, Oscar Gorria Cardesa

**Affiliations:** 1 Department of Urology, Navarra University Hospital, Pamplona, Spain; 2 Department of Urology, Marqués de Valdecilla University Hospital, Santander, Spain; 3 School of Medicine, University of Cantabria, Santander, Spain; 4 IDIVAL Research institute, Santander, Spain

**Keywords:** Urethral Stricture, Reconstructive surgical procedures, Patient reported outcomes, Cystoscopy

## Abstract

**Purpose:**

Despite modern advances in urethroplasty, little consensus exists on defining treatment success. The absence of reintervention was the main benchmark. However, recent efforts incorporate anatomical, functional and patient-reported criteria. Our aim is to evaluate urethroplasty outcomes using these four criteria, and to assess the progression of asymptomatic anatomical recurrences to predict the need for future treatments.

**Methods:**

An ambispective study was conducted on patients > 18 years undergoing anterior urethroplasty between 2020 and 2023. Minimum follow-up of 2 years. Techniques included anastomotic and augmentation urethroplasties. Follow-up performing urine culture, uroflowmetry, self-administered questionnaires, and endoscopic/radiological imaging. Success was defined as: anatomical (passage of a 17Ch cystoscope or normal urethrogram), functional (Qmax > 10 mL/s), asymptomatic (no urinary symptoms or infections), no need for further treatments, and overall success.

**Results:**

138 patients were followed for a mean of 35 months (IQR = 24.4–63.8). Success rates were: anatomical 68.8%, functional 71.7%, asymptomatic and retreatment-free 79%, and overall success 63.8%. USS-PROM and quality of life scores improved significantly with high satisfaction levels and maintained IIEF-5 scores. Among the 43 patients with anatomical recurrences, 22 were asymptomatic (51.2%) when re-stricture was identified. After 35 months, 16 out of 22 (72.7%) remained symptom-free. All symptomatic progressions, leading to further interventions, developed during the first 18 months.

**Conclusions:**

Anatomical and functional criteria result in lower success rates than symptoms-based or retreatment definitions. Most patients with asymptomatic anatomical strictures who do not develop symptoms in the first 18 months remain stable and do not require secondary treatments.

## Introduction

When defining the success rate after an urethroplasty surgery there is still some degree of controversy. The absence of reintervention has always been the most clinically meaningful endpoint, as emphasized by the recent YAU Reconstructive Working Group and the stricture-fecta consensus [1]. However, this criteria does not take into account the asymptomatic recurrences, nor the fact that some patients are unwilling to undergo further treatments [2].

Some groups suggested using a multiple outcome reporting approach as the most optimal way of presenting results after urethroplasty surgeries [3]. Indeed, recent studies have started incorporating anatomical, functional and patient-reported criteria demonstrating a wide range of patency rates depending on the criteria used [4]. However, despite these efforts to achieve an agreement on reporting outcomes, there is still a large variability between the published results, which makes them difficult to compare.

In addition, no consensus exists on the best follow-up protocol following urethral surgery, regarding for how long and how to evaluate the patients, but it seems reasonable that follow-up should last at least 12 months [5]. The group of Trauma and Urologic Reconstructive Network of Surgeons (TURNS) in 2014 established a follow-up after urethroplasty with routinely performed cystoscopies observing that one third of anatomical recurrences were asymptomatic after one year [6]. Further follow-up of asymptomatic anatomical recurrences demonstrated an increased risk of need for treatment [7], but there is still a need for tools for predicting which patients would need interventions.

We have evaluated long-term outcomes of patients following urethroplasty surgeries using four different criteria for success (anatomical, functional, patient-reported, and need for reintervention), and have assessed the progression of asymptomatic anatomical recurrences in order to predict the need for future treatments.

## Materials and methods

### Study population

We conducted an ambispective study in adult patients (≥ 18 years) who underwent anterior urethroplasty surgery in the University Hospital of Navarra between 2020 and 2023. The study followed the Good Clinical Practice guidelines and the principles of the Declaration of Helsinki. The study protocol was reviewed and approved by the Navarra Research Ethics Committee (CEIm) on July 15th, 2024 (approval code CEIm 2024/49).

Patients included should have at least 2 years of follow-up after urethroplasty surgery. We included only male patients, who underwent open surgeries for anterior urethral strictures -including anastomotic urethroplasties using transecting or non-transecting techniques and augmented urethroplasty using buccal mucosa grafts (BMG) or preputial grafts. Patients with an incomplete follow-up time, who underwent perineal urethrostomy, or had surgeries for posterior urethral stenosis (pelvic fractures or re-do vesico-urethral anastomosis) were excluded.

### Follow-up protocol

The follow-up assessments included urine cultures, uroflowmetry, and questionnaires at 3, 6, 12, 24 and 60 months postoperatively. In addition, we performed a urethro-cystoscopy -using a flexible 17 F cystoscope- or a retrograde urethrogram (RUG) at 6, 24 and 60 months to all patients.

The patients were asked to collect a urine culture 1 week before the visit, therefore, the day of the visit the culture was checked. During the same clinical appointment, the patients were asked to complete two questionnaires (self-administered): Spanish validated versions of Urethral Stricture Surgery Patient-Reported Outcome Measure (USS-PROM) [8] and International Index on Erectile Function (IIEF-5) [9]. Uroflowmetry and either RUG or urethro-cystoscopy were performed during the same appointment. The choice between the two tests for assessing anatomical success was offered to the patient.

The USS-PROM questionnaire was scored using 3 values: a first item calculated from the addition of questions 1 to 8 (minimum score 8); a second item “quality of life” (QoL) which ranges from 0 (worst health status imaginable) to 100 (best health status imaginable); and a third item “satisfaction” where 1 means “very satisfied”, 2 is “satisfied”, 3 is “unsatisfied” and 4 is “very unsatisfied” [10]. For erectile function assessment the patient completed the IIEF-5 questionnaire, getting a final score between 5 and 25.

### Definition of success

We evaluated urethroplasty success based on four criteria: (1) Anatomical success: passage of a flexible cystoscope 17Ch with no resistance, or lack of any signs of recurrence in the RUG. (2) Functional success: uroflowmetry with a maximum flow rate (Qmax) over 10 ml/s, performed with a voided volume of > 120 mL. (3) Symptoms: no sign of urinary infection in urine culture, or complain about low urinary stream either on self-administered questionnaires or during direct clinical interview with the patient. (4) No need of any further procedure, including placing indwelling urethral or suprapubic catheters, urethral dilatation, internal urethrotomy, or repeated urethroplasty. We added a final overall success percentage, defined by not presenting any of the previous failure criteria.

Published literature applied a broad range of Qmax thresholds to define functional success - using cut-off values of 15 mL/s or 12 mL/s, as recommended by the EAU [5]. However, uroflowmetry results may be influenced by operator variability, benign prostatic obstruction (BPO)/lower urinary tract symptoms (LUTS), bladder dysfunction, and differences in bladder capacity evidence. Furthermore, studies indicated that up to 20% of patients undergoing urethroplasty fail to reach Qmax of 14 mL/s despite having a patent urethra [11]. For these reasons, we adopted a less strict definition of functional success, defining it as a Qmax greater than 10 mL/s.

### Data collection

Demographic data (age, diabetes mellitus, arterial hypertension, Chronic Obstructive Pulmonary Disease (COPD), smoking), stricture data (stricture etiology, location in the anterior urethra, and stricture length) and previous surgeries were collected from patient hospital charts. Baseline questionnaires and flowmetry values were obtained and recorded during preoperative workout.

### Statistical analysis

Descriptive analysis was performed for main clinical and surgical variables. Quantitative variables were assessed for normality using Shapiro-Wilk test, and therefore described as mean ± standard deviation (SD), or median and interquartile range (IQR) and qualitative variables as number (percentage). Survival analysis of urethroplasty accumulative incidence of failure was expressed by a Kaplan-Meier curve. Analysis was conducted using STATA 13 software (StataCorp, College Station, TX, USA) for Mac.

## Results

A total of 138 patients were included in the study with a mean follow-up of 35 months (IQR = 63.8–24.4). The mean age was 58.6 years (SD = 14.9) with 15.2% of the patients presenting with diabetes, 29.7% with hypertension and 2.2% with COPD. Regarding smoking status, 17.4% were current smokers and 35.5% were previous smokers. Table 1 summarizes the descriptive characteristics of the study population.

The most frequent locations of stricture were bulbar (73.9%), followed by fossa navicularis and meatus (11.6%), penile (9.4%), bulbo-membranous (2.9%) and multiple locations in the anterior urethra (2.2%). Iatrogenic strictures were 60.1% of all causes, 1.5% had received previous radiation, and 9.4% presented with lichen sclerosus. Almost half of the patients underwent previous surgeries (49.3%), including urethrotomy, dilatations with a non-coated balloon, and previous urethroplasty. The length of the stricture was longer thar 20 mm in almost 56% of the patients.

We conducted 122 augmented urethroplasties with BMG, 7 augmented urethroplasties with preputial graft and 9 anastomotic urethroplasties (5 of them non-transecting and 4 classical excision and primary anastomosis).

**Table 1:** Demographical and clinical characteristics of 157 study participants

No. patients	138
Follow-up (Mean ± SD) months	35.3 ± 14
Age (Mean ± SD) years	58.7 15
No. diabetes cases (%)	21 (15.2%)
No. hypertension cases (%)	41 (29.7%)
No. COPD cases (%)	3 (2.2%)
*No. smoking history* (%)	
Never smoked	65 (47.1%)
Current smoker	24 (17.4%)
Previous smoker	49 (35.5%)
*No. stricture locations* (%)	
Penile	13 (9.4%)
Bulbar	102 (73.9%)
Bulbo-membranous	4 (2.9%)
Multiple locations	3 (2.2%)
Meatus and fossa navicularis	16 (11.6%)
*No. stricture etiology* (%)	
External trauma	8 (5.8%)
Iatrogenic	83 (60.1%)
Infectious	10 (7.2%)
Lichen sclerosus	13 (9.4%)
Idiopathic	11(8%)
Radiation	2 (1.5%)
Hypospadias	11 (8%)
*No. stricture length * %	
Stricture ≤ 20 mm	61 (44.2%)
Stricture > 20 mm	77 (55.8%)
*No. previous surgeries (%)*	69 (49.3%)
*No. repair type (%)*	
Anastomotic urethroplasty	9 (6.5%)
Augmented urethroplasty with BMG	122 (88.4%)
Augmented urethroplasty with preputial graft	7 (5.1%)

*COPD* Chronic obstructive pulmonary disease, *BMG* Buccal mucosa graft

**Fig. 1:**
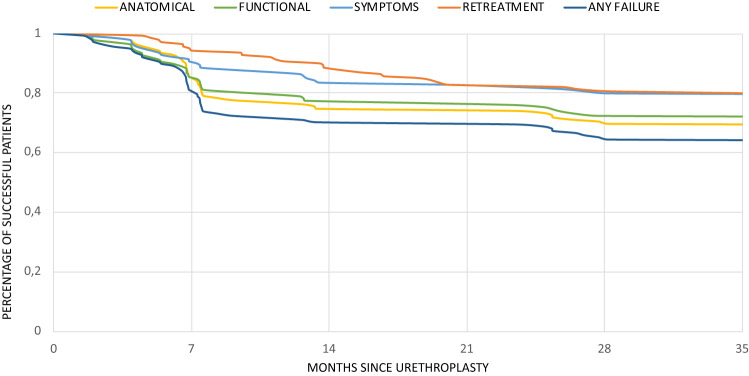
Probability of success of anterior urethroplasty according to different definitions

The success rates considering the four criteria were: 68.8.% for anatomical success, 71.7% for functional success, 79% for both symptoms-free and lack of retreatment and 63.8% for overall success. (Fig. 1).

A progressive improvement was observed in USS-PROM score compared to preoperative values. Scores in the first item decreased from 23.1 average points to 15.8 at the first interview (3 months) and remained stable even at 60 months follow-up. The QoL improved by 7–8 mean points (a 14% of improvement in the QoL) and reached the highest score in those patients with a complete follow-up of 5 years. Mean satisfaction ranged between “satisfied” and “very satisfied.” (Table 2). In our sample not many patients were sexually active, but the mean IIEF-5 score remained stable after surgery with a mean punctuation between 18 and 19 points.

**Table 2:** USS-PROM and IIEF-5 questionnaire scores at 3, 6, 12, 24 and 60 months

Timepoint	USS-PROM 1–8 (Mean ± SD)	PROM-QoL (Mean ± SD) (Mean SD)	PROM-Satisfaction (Mean ± SD)	IIEF-5 (Mean ± SD)
Preoperative	23.1 (± 5.5) *N* = 102	68.5 (± 19.1) *N* = 103	–	16.7 (± 7.4) *N* = 61
3 months	15.8 (± 5.3) *N* = 120	76.8 (± 16.9) *N* = 120	1.6 (± 0.6) *N* = 120	18.2 (± 7) *N* = 68
6 months	15.6 (± 5.3) *N* = 125	77.8 (± 14.8) *N* = 117	1.6 (± 0.6) *N* = 118	19.4 (± 6) *N* = 67
12 months	15.3 (± 4.4) *N* = 108	76.9 (± 17.1) *N* = 105	1.5 (± 0.6) *N* = 106	18 (± 5.2) *N* = 43
24 months	15.3 (± 4) *N* = 95	77.7 (± 17.9) *N* = 87	1.4 (± 0.5) *N* = 91	19.1 (± 6.6) *N* = 58
60 months	17 (± 4.3) *N* = 18	84.8 (± 10.5) *N* = 18	1.4 (± 0.5) *N* = 18	19.1 (± 6) *N* = 9

**Fig. 2:**
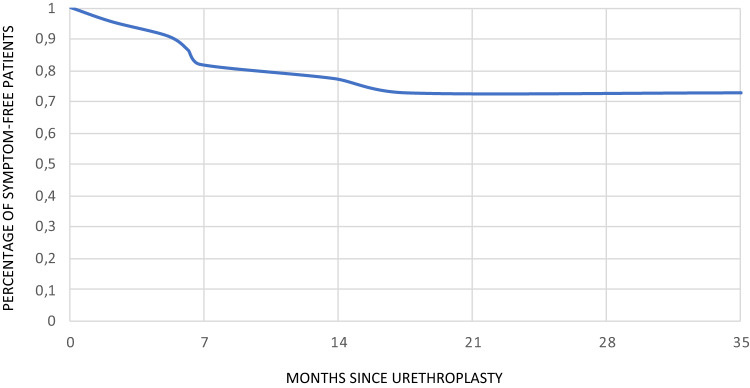
Time to development of symptoms in patients with initially asymptomatic anatomical strictures

A total of 43 patients had anatomical failure (31.2% of all urethroplasties), of whom 22 (51.2%) were asymptomatic at the time of the diagnosis of the recurrence of the stricture. Among these asymptomatic anatomical recurrences, 16 out of 22 patients (72.7%) remained asymptomatic after 35 months of follow-up and those who developed symptoms, did so within the first 18 months after the surgery (Fig. 2).

Among the patients who had anatomical recurrences, 67.4% (29 of 43 patients) undergone any reintervention; 11 patients underwent a re-do urethroplasty, 6 patients underwent a single urethral dilatation or entered a self-catheterization program, 6 patients had a paclitaxel-coated balloon dilatation, 5 patients underwent an internal urethrotomy, and one patient ended up with indwelling urethral catheter.

## Discussion

We present the outcomes of urethroplasty, with of a minimum follow-up of 2 years, in a large cohort of patients, specifically focusing on the need for reintervention in selected patients with asymptomatic anatomical recurrences. The results of our study confirm that using more objective measures such as urethro-cystoscopy, or uroflowmetry turns on a worse patency rate after urethroplasty - around 70% after 35 months- in contrast with more subjective criteria such as symptoms reported by the patient or undergoing a reintervention, which shows a success rate of almost 80%. These results are consistent with the ones presented by Anderson et al. [4] with a 5 year probability of success of 71% for anatomical, 58% for uroflow considering a Qmax > 15 ml/s, 75% for retreatment, and 37% for low urinary stream on questionnaires. The overall success rate in our study was 63.8% at 35 months in contrast with a 23% of success rate at 5 years reported by Anderson et al. [4]. Previous studies have applied a broad range of maximum flow rate (Qmax) thresholds to define functional success, most commonly using cut-off values of 15 mL/s or 12 mL/s, as recommended by the EAU [5]. However, evidence indicates that nearly 20% of patients undergoing urethroplasty fail to reach a Qmax of 14 mL/s despite having a patent urethra, particularly following a two-sided dorsal approach [11]. Moreover, uroflowmetry results may be influenced by operator variability, benign prostatic obstruction (BPO)/lower urinary tract symptoms (LUTS), bladder dysfunction, and differences in bladder capacity. For these reasons, we adopted a less stringent definition of functional success, defining it as a Qmax greater than 10 mL/s. We recognize that this lower threshold may lead to higher reported success rates compared with studies using a Qmax > 15 mL/s, but taking into considerations the mean age of our population, and the probability that apart from the urethral stricture, the bladder outlet obstruction could also be related with BPO, we decided to be less restrictive. Indeed, even in some cases with anatomical success we observed a functional failure as these patients could not achieve a Qmax > 10 ml/s. These criteria may explain the differences for functional success rate between our results and the ones presented by Anderson et al. (71% vs. 58%)

When considering the need of secondary interventions for treating recurrences after urethroplasty, it is increasingly apparent that not all strictures are accompanied by symptoms and, what is more, not all of them require reoperation -as happens with incidental strictures found during routine follow-up for bladder tumours [12]. The TURNS group in 2014 performed a follow-up with cystoscopy to all urethroplasties, with a 1-year anatomical success rate of 77.5%. Surprisingly, one third of those anatomical recurrences found by cystoscopy where asymptomatic at the time of the diagnosis [6]. Years later, this same group presented the outcomes of the same cohort after a longer follow-up, where only 53.3% of < 17Ch recurrences underwent a new intervention [13]. Along the same lines, Amend et al. in 2023 presented comparable results of 304 urethroplasty patients where 16.4% of patients presented < 17Ch recurrences, and of these 64% had a reintervention [7]. In our population 31.2% of patients had a < 17Ch recurrence (43 patients) and 67.4% of them (29 patients) required further treatment.

It is suggested that despite the recurrences, QoL scores between patients with anatomical recurrences and normal lumen are comparable, and even some patients are barely symptomatic, emphasizing the need for taking into consideration patient’s symptoms when considering reintervention [13]. We also consider necessary to discuss these aspects when explaining the success rates of urethroplasty in the preoperative stage [1].

Our study presents some limitations as its retrospective nature, and the heterogeneity of anterior urethral strictures and urethroplasty techniques included. On the other hand, exclusion of patients who underwent perineal urethrostomy, which we consider as a definitive form of urinary diversion rather than a reconstructive procedure intend to re-establish urethral continuity, reduces confusion with interpretation of treatment success. Our strict follow-up program, collecting prospectively the subjective data using self-administered validated questionnaires also make the conclusions obtained from them more consistent. Furthermore, collecting only patients with intermediate to long-term follow-up allows for describing the course of the disease with a longitudinal perspective.

What is most interesting about our study is describing the natural history of the patients with asymptomatic recurrences. Our results confirm the fact that, when diagnosing an anatomical recurrence, if no symptoms are associated, it is worthwhile to monitor these patients as only 28% of them will progress to a required treatment in the first 18 months, and almost all of the rest will remain stable, at least, after 35 months of follow-up.

## Conclusions

Anatomical and functional criteria result in lower success rates than symptoms-based or retreatment definitions. Most patients with asymptomatic anatomical strictures who do not develop symptoms in the first 18 months will remain stable and will not require further intervention during long-term follow-up.

## Data Availability

The data are not publicly available due to ethical and/or legal restrictions but may be made available upon reasonable request and with approval from the appropriate ethics committee.
